# Modified Composite Based on Magnetite and Polyvinyl Alcohol: Synthesis, Characterization, and Degradation Studies of the Methyl Orange Dye from Synthetic Wastewater

**DOI:** 10.3390/polym13223911

**Published:** 2021-11-12

**Authors:** Cristina Modrogan, Simona Cǎprǎrescu, Annette Madelene Dǎncilǎ, Oanamari Daniela Orbuleț, Alexandru Mihai Grumezescu, Violeta Purcar, Valentin Radițoiu, Radu Claudiu Fierascu

**Affiliations:** 1 Department of Analytical Chemistry and Environmental Engineering, Faculty of Applied Chemistry and Materials Science, University Politehnica of Bucharest, Ghe. Polizu Street No 1-7, 011061 Bucharest, Romania; cristina.modrogan@upb.ro (C.M.); annette.dancila@upb.ro (A.M.D.); oanamari.orbulet@upb.ro (O.D.O.); 2 Inorganic Chemistry, Physical Chemistry and Electrochemistry Department, Faculty of Applied Chemistry and Materials Science, University Politehnica of Bucharest, Ghe. Polizu Street No 1-7, 011061 Bucharest, Romania; 3 Department of Science and Engineering of Oxide Materials and Nanomaterials, Faculty of Applied Chemistry and Materials Science, University Politehnica of Bucharest, Ghe. Polizu Street No 1-7, 011061 Bucharest, Romania; alexandru.grumezescu@upb.ro (A.M.G.); fierascu.radu@icechim.ro (R.C.F.); 4 Research Institute of the University of Bucharest—ICUB, University of Bucharest, 050657 Bucharest, Romania; 5 Academy of Romanian Scientists, Ilfov No. 3, 50044 Bucharest, Romania; 6 National Institute for Research and Development in Chemistry and Petrochemistry—ICECHIM, Splaiul Independentei, No. 202, 060021 Bucharest, Romania; violeta.purcar@icechim.ro (V.P.); vraditoiu@yahoo.com (V.R.)

**Keywords:** modified composite, magnetite, polyvinyl alcohol, methyl orange, degradation, wastewater

## Abstract

The goal of the present paper was to synthesize, characterize, and evaluate the performance of the modified composite based on magnetite (Fe_3_O_4_) and polyvinyl alcohol (PVA). The obtained composite was used to degrade Methyl Orange dye from synthetic wastewater by a laboratory photocatalytic reactor. Various parameters of the photodegradation process were tested: composite dosage, amount of hydrogen peroxide (H_2_O_2_), and pH. The composite was characterized by Fourier Transform Infrared (FTIR) Spectroscopy, X-ray Diffraction (XRD), and Scanning Electron Microscopy (SEM). The degradation experiments indicated that the complete dye decolorization depended on the amount of H_2_O_2_. In addition, the H_2_O_2_ could accelerate Methyl Orange degradation to more highly oxidized intermediates in the presence of UV light (99.35%). The results suggested that the obtained modified composite could be used to treat wastewater containing various types of dyes.

## 1. Introduction

Currently, the elimination of harmful industrial dyes from wastewater is of utmost interest, as the quality of drinking water in the world is steadily declining. As these pollutants discharged into the water are largely constituent elements of various effluents from various industries (e.g., pharmaceutical, cosmetic, and food production), it is necessary to use advanced effluent treatment methods before discharging them into the environment [1,2]. Industries that produce textiles, paper, plastics, leather, nutriments, and cosmetic products use various dyes widely [1,3,4]. Dye-containing effluents resulting from these types of industries are released into water, and this action constitutes a substantial threat to aquatic life, as well as to the environment [3,5]. The water-soluble azo dye Methyl Orange has a highly carcinogenic risk factor and is extensively used in textile industries, manufacturing printing paper, and research laboratories. Methyl Orange has the capacity of also being metabolized into aromatic amines by intestinal microorganisms [3]. This dye, which is difficult to eliminate from aqueous solutions by common water purification/treatment methods because of its solubility in water, is also stable, with a low biodegradability [5].

In recent years, advanced oxidation processes (AOPs) [1,3,6] have attracted greater interest in the treatment of wastewater that contains dyes, in comparison with other methods such as ozonation, biodegradation, chlorination, and biological methods [1,2,3], due to its advantages: high efficiency, total destruction of organic pollutants using active photocatalysts in a few hours at room temperature, and cost-effectiveness [7,8,9,10]. Among the AOPs, the photodegradation process of organic dyes using composites based on unmodified or modified magnetite (Fe_2_O_3_) has been applied for the degradation of the different dyes (e.g., Acid Red73 [8], Methylene Blue [9,10,11], Malachite green [12], Crystal Violet [13]). Golshan et al. [3] found that the synthesized magnetite hydroxyapatite nanoparticles (Fe_3_O_4_@HAP) had a good performance in the degradation of Acid Red73. The results showed that the degradation efficiency of the dye was around 97% under UV irradiation, for 1 h. Chen et al. [8] reported that the Fe_3_O_4_/Ag_6_Si_2_O_7_ composites, fabricated by the precipitation process, can be used to remove Methylene blue dye from water under simulated visible light. They found that the removal efficiency of dye was higher (98%). Guidolin et al. [10] indicated that the magnetite nanoparticles can be used to degrade Methylene blue dye from a simulated aqueous solution. The results showed that the most effective color removal of 93.4% was obtained when a higher concentration of nanoparticles of 2250 mg·L^−1^ was used in 210 min. Rivera et al. [11] studied the Methylene blue (MB) dye degradation using Fe_3_O_4_ nanoparticles by the Fenton-Like process. They found that a 100% color degradation was obtained for 2 g·L^−1^ of Fe_3_O_4_ nanoparticles and a concentration of 100 mg MB/L at pH 3.5. Arifin et al. [12] synthesized Fe_3_O_4_/TiO_2_/CuO nanocomposites for the degradation of Malachite green from aqueous solution under UV and visible light irradiation. The results suggested that the photocatalytic activity of nanocomposites was enhanced under visible light irradiation.

The synthesis of Fe_3_O_4_ nanoparticles containing polymers (e.g., poly(vinyl alcohol) (PVA), poly(vinylpyrrolidone) (PVP)) has rarely been reported in the literature [14,15,16]. Mahmoudi et al. [14] prepared magnetite nanorods using polyvinyl alcohol (PVA) using the co-precipitation method. They reported that the formation of magnetic beads was favored when a higher concentration of PVA (polymer/iron mass ratio of 5) was used. Seo et al. [15] reported the synthesis of Fe_3_O_4_ using PVP. They reported that PVP protects the Fe_3_O_4_ powder from further oxidation and prevents the agglomeration of the Fe_3_O_4_. Usawattanakul et al. [16] prepared the nanocomposite film of poly(vinyl alcohol) (PVA) incorporated with bacterial cellulose nanocrystals and magnetite nanoparticles (Fe_3_O_4_) by the in situ synthesis technique using chemical coprecipitation. Their study showed that the prepared film, due to its high hydrophilicity, can be applied in diverse fields for the adsorption of various pollutants.

In this work, we prepared unmodified and modified composites based on magnetite (Fe_3_O_4_) and polyvinyl alcohol (PVA) by the co-precipitation method. These composites were prepared for the removal of Methyl Orange dye from synthetic wastewater using a laboratory photocatalytic reactor under various conditions. The composite was characterized by Fourier Transform Infrared (FTIR) Spectroscopy, X-ray Diffraction (XRD), and Scanning Electron Microscopy (SEM). Influencing factors regarding photocatalytic degradation such as composite dosage, amount of hydrogen peroxide (H_2_O_2_), and pH value were also investigated. In addition, the effect of UV irradiation on the Fe_3_O_4_/PVA composite was studied.

## 2. Experimental Methods

### 2.1. Materials and Methods

Ferrous sulfate hexahydrate (Fe(SO_4_)·6H_2_O)) and ferric chloride (FeCl_3_) were purchased from Chimopar (Chimopar SRL, Bucharest, Romania). Ammonia (NH_3_) and poly(vinyl alcohol) (PVA) were purchased from Sigma Aldrich (Merck KGaA, Darmstadt, Germany) without additional purification. Hydrogen peroxide (H_2_O_2_), sulfuric acid (H_2_SO_4_), and manganese dioxide (MnO_2_) were purchased from Merck (Merck KGaA, Darmstadt, Germany). All reagents that were used were of analytical grade. The distilled water was also used in this study to prepare the aqueous solution of dye.

Methyl Orange powder (anionic, water-soluble azo dye) was purchased from Sigma Aldrich (Merck KGaA, Darmstadt, Germany) and was utilized without any additional purification. The stock solution of Methyl Orange was prepared by dissolving powder of Methyl Orange in deionized water at room temperature (23 ± 2 °C) to obtain a solution concentration of 10 × 10^−4^ mol·L^−1^. The working solution was prepared by diluting the stock solution with deionized water (5 × 10^−4^ mol·L^−1^) and sulfuric acid (H_2_SO_4_). The sulfuric acid was used to adjust the pH of the solution at 3, measured using a pH-meter JK-PH009 (Shanghai Jingxue Science Apparatus Co., Ltd., Shanghai, China). An amount of the concentrations above was utilized to calibrate a curve of absorbance against concentration at a prearranged wavelength at a maximum absorbance λ = 464 nm utilizing a UV-Visible spectrophotometer Shimadzu 9100 (Shimadzu Scientific Instruments, Columbia, SC, USA). The sensitivity and accuracy of the machine were maximally improved using measurements of absorbance at a wavelength. From this calibration curve, we could establish the exact concentrations.

### 2.2. Synthesis of the Composite

Magnetite (Fe_3_O_4_) was obtained directly by the precipitation reaction between prepared solutions of FeSO_4_ 0.2 mol·L^−1^ and of FeCl_3_ 0.2 mol·L^−1^ with concentrated ammonia (NH_3_ 25%) without heat treatment.

The Fe_3_O_4_/PVA composite was prepared by the following procedure: in a Berzelius beaker, the necessary volumes (in stoichiometric quantities of 200 mL) of the prepared solutions (FeSO_4_ and FeCl_3_) were added in order to obtain 3 × 10^−3^ g of Fe_3_O_4_. Under continuous mechanical stirring (400 rpm) (RSLAB 13 PRO 20 Digital Mechanical Stirrer, Distribution Account Manager Laboratory, Telecomed, Iasi, Romania; IKA^®^-Werke GmbH & Co. KG, Deutschland, Germany), the concentrated ammonia (15 mL) was added over the obtained solution. Afterward, the mixture was stirred for another 20 min (at 400 rpm) until a black precipitate was obtained. The solution above the precipitate (immobilized with a magnet placed under the beaker) was decanted. The obtained precipitate was washed three times with 50 mL of distilled water until the chloride ions were completely removed. Then, a PVA solution (5%) was added over the mix of precipitate formed and was magnetically stirred together at 400 rpm for 20 min at room temperature (23 ± 2 °C). After the mixing time had elapsed, the solution was filtered and rinsed with distilled water and was then transferred to a watch bottle (weighed beforehand) for drying. The formed precipitate was slowly heated at 100 °C, using an oven (Multilab ML-LE 15/11, Distribution Account Manager Laboratory, Analytical Equipment MultiLab SRL Romania, Bucharest; IKA^®^-Werke GmbH & Co. KG, Deutschland, Germany). After gelling, the temperature was increased to 150 °C and the precipitate was heated for another 4 h using the oven in order to obtain the Fe_3_O_4_/PVA composite. The PVA was used for the preparation of Fe_3_O_4_/PVA (modified composite), because the PVA molecules are rich in OH^−^ functional groups, which enable them to participate in the formation of magnetic beads.

**Figure 1:**
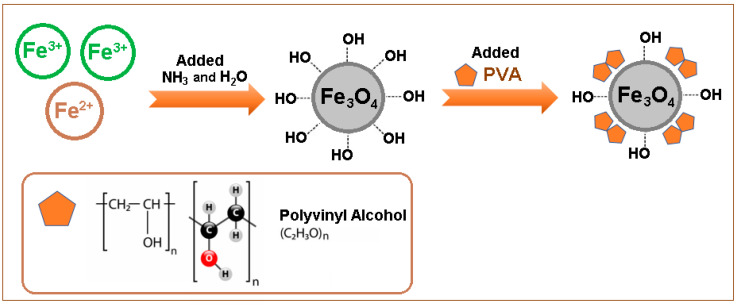
Schematic representation of the preparation of Fe_3_O_4_/PVA (modified composite).

The schematic preparation of Fe_3_O_4_/PVA (modified composite) is illustrated in Figure 1.

The obtained composites (Fe_3_O_4_ andFe_3_O_4_/PVA) were crushed before being characterized and used for the photocatalytic tests.

### 2.3. Photocatalytic Experimental Procedure

**Figure 2:**
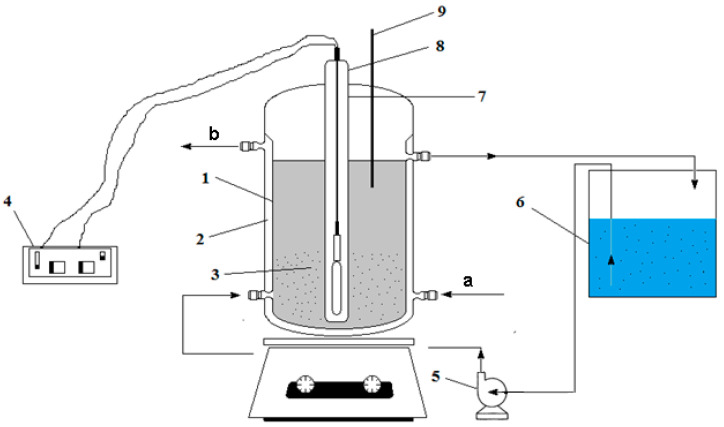
Laboratory installation composed of the following elements: 1—photocatalytic reactor; 2—cooling jacket (a, b); 3—Fe_3_O_4_ or Fe_3_O_4_/PVA (modified composite); 4—UV lamp source; 5—recirculation pump; 6—recirculation reservoir; 7—UV lamp; 8—quartz tube; 9—thermometer.

The experiments were performed in a laboratory installation using a photocatalytic reactor shown in Figure 2. The photocatalytic reactor had the following dimensions: inner diameter of the reactor, 80 mm; outer diameter of quartz tube, 35 mm; intertubular distance (irradiation), 23 mm. The photocatalytic reactor had the following characteristics: useful volume (reaction), 1.5 L; total solution volume, 2 L; pump flow, 1 L·min^−1^; contact time (when recirculating), 1.5 min.

The degradation of the organic compounds was performed in a laboratory installation with continuous recirculation, using a cylindrical reactor with reaction space (1) with a jacket on which the cooling water flowed (2). The photocatalytic reactor, composed of a UV radiation generator (4), of the Hg vapor quartz lamp type (8) was positioned axially centrally in a quartz tube to form an annular space reaction (7). Fe_3_O_4_ or Fe_3_O_4_/PVA (3) was inserted around the quartz tube. The wastewater-containing dye was introduced into the reactor from a tank (6) by means of a pump (5). The working temperature of synthetic wastewater was maintained constant at 20 °C using a thermometer (9).

The samples were analyzed at a wavelength of 464 nm using a UV-Vis spectrophotometer (Shimadzu 9100, Shimadzu Scientific Instruments, Columbia, SC, USA). The samples gathered from the reactor at distinct reaction times were stabilized through the amount of MnO_2_ addition for fast decomposition of the unreacted H_2_O_2_. The pH was measured using a pH meter.

The tests on the degradation of the organic compounds from prepared wastewater were realized at 20 °C utilizing a photo-assisted procedure (H_2_O_2_ + UV). The experiments were conducted only in the presence of the Fe_3_O_4_/PVA, to show the effect of PVA incorporated into Fe_3_O_4_.

### 2.4. Characterization of the Composites

FTIR spectroscopy was used to highlight the formation of magnetite in the synthesized samples. FTIR spectra were recorded using an FTIR spectrometer (Jasco FTIR 6300, JASCO Int. Co., Ltd., Tokyo, Japan) equipped with a Golden Gate Specac ATR (KRS5 lens), in the range of 4000–400 cm^−1^ (32 scans for each point at a spectral resolution of 4 cm^−1^). The deconvolution of the FTIR spectra was carried using the Jasco spectrum analysis software program (Spectra Manager II software from Jasco Inc., Tokyo, Japan).

The evaluation of the phase composition of the samples was performed using X-ray diffraction analyses, with a Rigaku SmartLab (Rigaku Corp., Tokyo, Japan), operated at 45 kV and 200 mA, with CuKα radiation (1.54059 Å), working in parallel beam configuration (2θ/θ scan mode), with the diffractograms being recorded in the range of 5–90° (2θ). The individual components were identified using the Rigaku Data Analysis Software PDXL 2 database provided by ICDD.

To investigate the morphology and dimensions of the nanostructured thin layers (obtained composites), the samples were sectioned using a diamond disc placed on a support and were introduced into an FEI Electron Microscope (SEM) (Hillsboro, OR, USA). The obtained images were recorded using secondary electron beams at an energy of 30 kV.

## 3. Results and Discussion

### 3.1. Characterization of Composites

**Figure 3:**
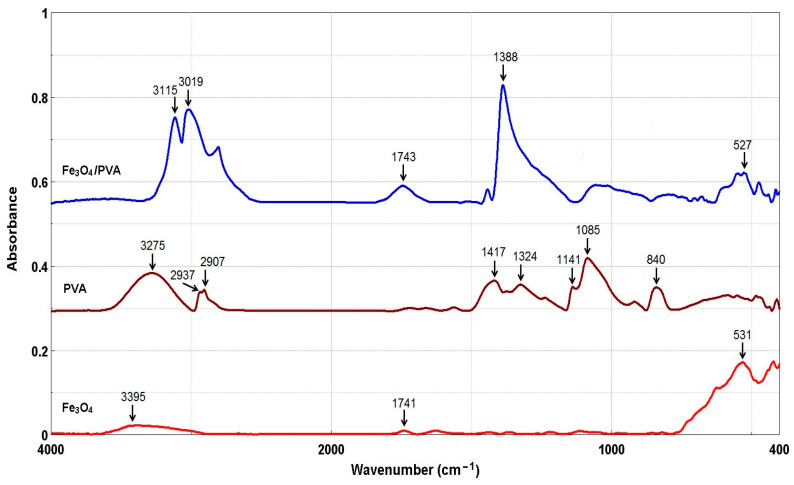
FTIR spectra of Fe_3_O_4_, PVA, and Fe_3_O_4_/PVA.

To confirm the formation of the Fe_3_O_4_, the original and modified magnetite (Fe_3_O_4_/PVA) were analyzed using FTIR spectroscopy. The FTIR spectra of samples are shown in Figure 3.

From Figure 3, for samples Fe_3_O_4_ and Fe_3_O_4_/PVA, the presence of a peak at ~530 cm^−1^ can be seen, which confirms the stretching vibrations of the Fe-O bonds [17,18,19]. The peaks detected at 1388 cm^−1^ (Fe_3_O_4_/PVA) and 1324 cm^−1^ (PVA) are related to methylene stretching [16,19,20,21,22]. The peak at 3275 cm^−1^ observed in PVA is ascribed to the bonded hydroxyl (O–H stretching) vibration. The absorption peaks at 2937 cm^−1^ and 2907 cm^−1^ are assigned to C–H asymmetric stretching [19,20,21]. The peaks observed at 1417 cm^−1^, 1085 cm^−1^, and 840 cm^−1^ are assigned to CH_2_ bending, C–O stretching, and C–C stretching, respectively [16,19,20,21,22,23]. The peak at 1141 cm^−1^ is characteristic of C–O bond stretching of the carboxylic groups [16,19,20,21]. In the Fe_3_O_4_ and Fe_3_O_4_/PVA spectra, the absorption peaks in the region of 3400–3000 cm^−1^ are assigned to the O–H stretching of hydroxyl groups [19,20,21]. The peak at ~1740 cm^−1^ is attributed to the stretching vibration of C–O. The changes observed in the Fe_3_O_4_/PVA in comparison with the Fe_3_O_4_ could be attributed to the chemical interactions between the functional groups of the PVA and Fe_3_O_4_.

**Figure 4:**
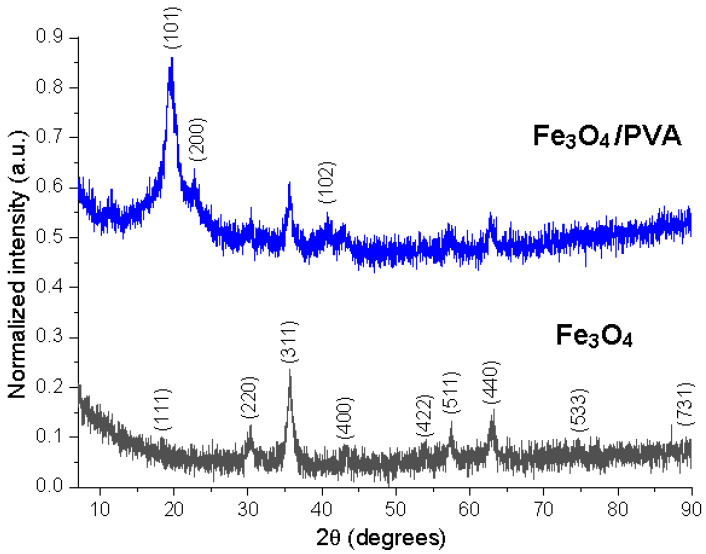
X-ray diffractograms of the Fe_3_O_4_ and Fe_3_O_4_/PVA—normalized intensities.

Figure 4 shows the normalized XRD patterns of the two samples. Normalization of the X-ray diffractograms allows the comparison of different materials, exceeding the limitations related to experimental variables [24]. The diffractograms correspond to cubic Fe_3_O_4_ by comparison with ICDD PDF card no. 01-088-0315, with diffractions peaks (111), (220), (311), (400), (422), (511), (440), (533), and (731). The Fe_3_O_4_/PVA composite material presented supplementary diffraction peaks, associated with PVA, at 19.2° (2θ) (a high-intensity broad peak), 22.9 (low-intensity shoulder peak), and 40.5 (low-intensity broad peak), corresponding to the diffraction planes (101), (200), and (102), respectively, marked with an asterisk on Figure 4 [25,26,27]. Alves et al. [28] synthesized the hybrid material of magnetic iron oxide (MOM) with citric acid-doped polyaniline, (PAni(CA)). They reported that a peak of low relative intensity was observed at 2θ = 25.6° in the PAni(CA)/MOM hybrid samples. Sanad et al. [29] prepared a magnetic photocatalyst from natural iron ores. They found that the crystallographic planes (220), (311), (400), and (440) of magnetite correspond to 2θ = 30.5, 35.5, 43, and 63°.

**Figure 5:**
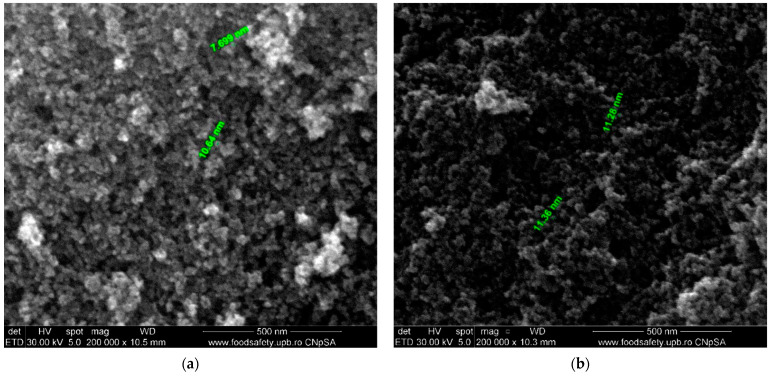
Scanning electron micrographs of (**a**) Fe_3_O_4_ and (**b**) Fe_3_O_4_/PVA.

The surface morphology of the prepared magnetite (Fe_3_O_4_) and modified magnetite (Fe_3_O_4_/PVA) was examined using SEM analysis (magnification 200,000×), which is shown in Figure 5.

It can be observed from the SEM images (Figure 5) that the Fe_3_O_4_ presented a nanometric size, with an average size of 9 nm (Figure 5a). It can also be observed that the Fe_3_O_4_ particles were stabilized by adding PVA to their surface and the Fe_3_O_4_/PVA particles had a relative diameter of about 11 nm (Figure 5b). Both types of nanoparticles seemed to be quasi-spherical-shaped [30,31]. It was reported in the literature that the small particles can be favorable for the adsorption of dye, and further, this can promote the photocatalytic reaction [8].

### 3.2. Photocatalytic Degradation

The experimental procedure of the photocatalytic tests involved mixing different amounts of the composite (1.5, 2, 3, and 5 g) with 2 L of aqueous solution of dye with a concentration of 5 × 10^−4^ mol·L^−1^. The mixture was recycled for 120 min to establish the equilibrium of photocatalysis between the dye and the surface of the composites.

It was established primarily by trial studies on how to decide the initial concentration of the operating solutions and to evolve an experimental scheme in correlation with a series of modifying factors (composite dosage, amount of H_2_O_2_, pH). Based on our preliminary data, a low amount of composite (<1.5 g) led to a low dye removal percentage.

The dye removal (%) was calculated using Equation (1) [29,32]:

(1) $$\mathrm{Dye}\;\mathrm{removal}(\%)=(1-\frac{C_{f}}{C_{\mathrm{in}}})\cdot 100,$$

where: C_in_—initial concentration of dye (mol·L^−1^); C_f_—final concentration of dye (mol·L^−1^).

After each test of the photodegradation, for the calculation of dye removal percentage, the procedure for the determination of the concentration of Methyl Orange from solutions was conducted in triplicate, and the obtained results were presented as means ± standard deviation. The analysis of concentrations was performed spectrophotometrically at λ = 464 nm.

#### 3.2.1. Effect of Composite Dosage on Dye Removal

The composite dosage is one of the important factors in the process of the photodegradation of organics because the optimal dosage of composite ensures more active sites and the effective absorption of photons. To investigate the influence of the modified composite dosage (Fe_3_O_4_/PVA) on Methyl Orange removal, a modified composite series of four dosages (1.5, 2, 3, and 5 g) was carried out, and the results are presented in Table 1. To determine the effect of the amount of composite on the removal efficiency of Methyl Orange, a synthetic solution with an initial concentration of Methyl Orange of 5 × 10^−4^ mol·L^−1^, at pH of 3.0, was used, with an initial amount of H_2_O_2_ (6.7 mL) and a contact time of 120 min.

It can be seen from Table 1 that with the increase in the amount of composite, the dye removal percentage value increased gradually. The dye removal percentage was significantly enhanced when the amount of Fe_3_O_4_/PVA was increased from 1.5 to 5 g. A higher removal percentage of dye (98.65%) was obtained for Fe_3_O_4_/PVA (5 g), possibly due to the combination of Fe_3_O_4_ with PVA. The values of dye removal percentage for Fe_3_O_4_/PVA were higher in comparison with the values obtained for Fe_3_O_4_, possibly due to the increasing amount of the composite and because the PVA molecules are rich in OH^−^ functional groups. This can lead to an increase in the adsorption sites available on the surface of Fe_3_O_4_/PVA and the active free radicals such as •OH generated from the activation of PVA by Fe_3_O_4_/PVA. Sanad et al. [29] investigated the influence of catalyst (argon-modified banded iron formation ore) dosage for the photocatalytic removal of methylene blue dye. The results showed that the photodegradation efficiencies after 120 min, at pH of 6.7, were 54.6%, 87.5%, and 84.8% at BIF doses of 0.25, 0.5, and 0.75 g L^−1^, respectively.

#### 3.2.2. Effect of the Amount of H_2_O_2_

The concentration of hydrogen peroxide plays a crucial role in deciding the overall efficiency of the degradation process. The effect of the amount of H_2_O_2_ was researched by evaluating the oxidation procedure. The amount of H_2_O_2_ was 3.5, 6.7, and 10 mL. Based on the preliminary data, we observed that the use of a higher excess (>10 mL) of H_2_O_2_ was sustained by a large concentration of H_2_O_2_ in the reaction medium, leading to a “scavenger” effect on the HO• and HO_2_• radicals, which expands with the growth in concentration, so the generation of radicals is limited significantly [8,29]. During the photocatalytic process, the active species such as hydroxyl radicals (HO• radical scavenger), superoxide radical anions (•O_2_^−^ radical scavenger), and holes (h^+^ radical scavenger) can be consumed by adding H_2_O_2_ [28,29]. In this research, an amount of H_2_O_2_ between 3.5 and 10 mL was used, to avoid the mentioned effects.

The results indicated in Table 2 show that the Methyl Orange photodegradation improved rapidly by increasing the amount of H_2_O_2_ (6.7 mL) followed by a decrease in photodegradation with a further increment in the amount of H_2_O_2_ (10 mL). The decreases in dye removal percentage can be explained by the higher amount of H_2_O_2_ resulting in a higher number of absorbed photons [3]. In addition, more photocatalytic sites can be available, leading to an increase in the dye removal percentage. However, care must be taken because the part of hydrogen peroxide not used during the degradation process is inhibited, and therefore, an excess amount is not recommended. It was reported in the literature that the presence of hydrogen peroxide is harmful to many organisms and will significantly affect the overall degradation efficiency if photocatalytic oxidation in the presence of Fe_3_O_4_ is used as a pre-treatment for biological oxidation. A negative effect of hydrogen peroxide is that of capturing the hydroxyl radicals generated. This occurs when large amounts of hydrogen peroxide are used [3,8].

#### 3.2.3. Effect of pH

**Figure 6:**
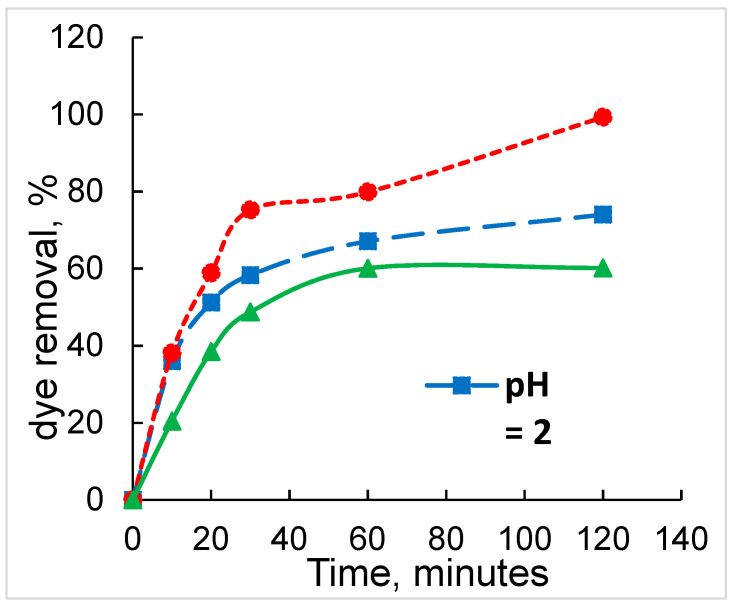
Effect of pH on the photocatalytic degradation of Methyl Orange under the following conditions: initial dye concentration of 5 × 10^−4^ mol·L^−1^, the concentration of H_2_O_2_ = 30%, and room temperature of 23 ± 2 °C.

In the photocatalysis process, the pH of the reaction medium plays a very important role. The effect of pH on the photodegradation of Methyl Orange in the presence of Fe_3_O_4_/PVA (5 g) at pH values between 2 and 4 is shown in Figure 6. At different times (10, 20, 30, 60, and 120 min), samples were taken once and analyzed to determine the dye removal percentage. The analysis was performed at λ = 464 nm.

The dye removal efficiency using the Fe_3_O_4_/PVA catalyst composite, at the initial pH values of 2, 3, and 4, was analyzed (Figure 6). The results showed that a higher dye removal efficiency (99.3%) occurred at a solution pH of 3, after a contact time of 120 min.

The estimation of the oxidation in time at distinct initial pH values is shown in Figure 6. As it is shown, under testing conditions, the degree of oxidation was affected by the initial pH of the solution, with the maximum estimation being acquired at a pH of 3. At lower pH values of the testing solutions (pH = 2), the degree of oxidation is reduced. This can be assigned to the operation of protonation of the molecules of H_2_O_2_ conducting to the formation of oxonium ions, ions that are much steadier toward oxidation, which is not engaging any more in the generation of HO• and HO_2_• radicals active in the oxidation [8,10]. It can be observed from Figure 6 that at values of pH of 2 and 4, respectively, dye removal was over 78% with 120 min of photodegradation in comparison to the photodegradation at pH of 3 where the dye removal was 99.3%. At pH = 4, the level of oxidation decreased in a stronger process owing to the precipitation of the Fe^3+^ ions to Fe(OH)_3_ and the development of complexes. The effect of the reaction intermediates is prone to their stability in relation to oxidation and their interaction with the catalytic element. The generation of HO• and HO_2_• radicals is restricted by the vanishing in the solution of the active centers related to the Fe^2+/3+^ ions and the absorption of the UV radiation [8,10,32]. Sanad et al. [29] studied the photocatalytic activity of the modified iron ore obtained by mixing the banded iron formation (BIF) sample with polyvinyl alcohol (PVA). The photocatalytic performance of modified BIF ore samples was examined to degrade the methylene blue (MB) dye at room temperature under UV irradiation. The results indicated that the BIF sample exhibited photocatalytic removals of 85.6%, 87.5%, and 74.3% at pH 4, pH 6.7, and pH 9 after 120 min, respectively, under UV irradiation. Elmorsi et al. [32] investigated the effect of initial pH. Solutions of Mordant red 73 (MR73) dye were irradiated at various initial pH (2, 3, 5, and 9). The results showed small differences in the degree of decolorization of the dye at different pH values. For example, in the case of 10 min reaction times, the decolorization efficiencies were 38.9%, 33.5%, 37.3%, and 35.4% at initial pH values of 2, 3, 5, and 9. At 50 min reaction times, the decolorization efficiencies changed slightly (94.3%, 98.9%, 94.5%, and 96.5%) for initial pH values of 2, 3, 5, and 9. The results indicated that MR73 degraded significantly at pH = 3, possibly due to the production of the corresponding weak acidic intermediates as a result of degradation and cleavage of the azo group in the dye.

#### 3.2.4. Effect of the Fe_3_O_4_/PVA Composite under UV Irradiation

**Figure 7:**
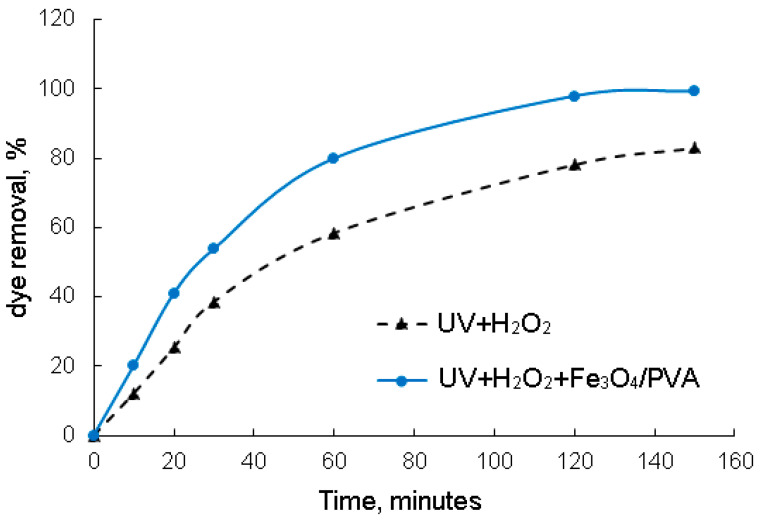
Evaluation of the photodegradation process as a function of the reaction time, at pH of 3 and initial dye concentration of 5 × 10^−4^ mol·L^−1^.

The effect of UV irradiation on the photodegradation of Methyl Orange in the absence and presence of Fe_3_O_4_/PVA (5 g), at pH of 3, is shown in Figure 7. At different times (10, 20, 30, 60, 120, and 150 min), samples were taken once and analyzed to determine the removal percentage of Methyl Orange.

The tests (UV + H_2_O_2_ vs. UV + H_2_O_2_ + Fe_3_O_4_/PVA) shown in Figure 7 highlight the relative scarce degradation process (83.05% in the case of the UV + H_2_O_2_ process and 99.35% in the case of UV + H_2_O_2_ + Fe_3_O_4_/PVA), indicating high stability toward oxidation of the organic compounds present in the wastewater effluent.

From Figure 7, it can be observed that the Fe_3_O_4_/PVA composite under UV irradiation led to a faster photodegradation of Methyl Orange dye. The removal of dye can be improved from 83.05% to 99.35% in 160 min. The presence of PVA in the prepared composite and H_2_O_2_ could accelerate the Methyl Orange degradation to more highly oxidized intermediates in UV light. This fact can indicate that the high dye removal efficiency is completely attributed to the photocatalytic performance of Fe_3_O_4_/PVA under UV + H_2_O_2_ [8,32,33]. Alves et al. [28] studied the photodegradation of the methylene blue dye in an aqueous solution under irradiation of ultraviolet light using the hybrids material of magnetic iron oxide (MOM) with citric acid-doped polyaniline (PAni(CA)). The results showed that the high reduction of the dye concentration was 98% in the presence of PAni(CA)/MOM (mass of aniline:MOM was 2:1). Elmorsi et al. [32] investigated the removal of Mordant red 73 (MR73) azo dye using H_2_O_2_/UV and the photo-Fenton reaction. The results indicated that the degradation of MR73 by the H_2_O_2_/UV process resulted in a 65% removal, and by the photo-Fenton reaction (H_2_O_2_/Fe^0^/UV), the highest efficiency was obtained for the degradation of MR73 dye with disappearance of about 99% in 15 min, indicating that the dominant decolorization mechanism is the photo-Fenton reaction.

#### 3.2.5. Photodegradation Mechanism

**Figure 8:**
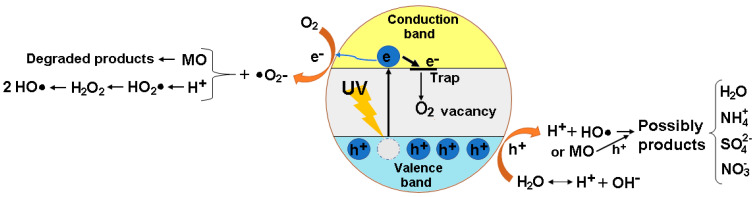
Proposed photodegradation mechanism of Fe_3_O_4_/PVA composite for the degradation of Methyl Orange (MO) dye.

Based on the obtained results from the photodegradation experiments of Methyl Orange dye using Fe_3_O_4_/PVA, the possible mechanism of dye under UV irradiation that can take place is illustrated in Figure 8.

It can be seen from Figure 8 that the photocatalytic activity depends on the capacity of the Fe_3_O_4_/PVA composite to create electron–hole pairs. Under UV irradiation, the photons were absorbed by the composite. After that, the electrons (h^+^) at the valence band were excited to the conduction band, creating the electron–holes in the valence band. The electron and the hole migrated to the composite surface and took part in the reactions shown in Figure 8. During the photodegradation process, the superoxide radical anions (•O_2_^–^) were generated by the reaction between O_2_ adsorbed on the surface of the composite and e−. The photogenerated holes can oxidize dye directly. In addition, the photodegradation of the dye can be achieved either using H_2_O_2_ or using H_2_O molecules and OH^−^ groups as electron donors to form free radicals (e.g., hydroxyl radicals: HO•) [3,8,33]. It was reported in the literature that the HO• and •O_2_^–^ are capable of degrading aromatic molecules [34,35,36].

## 4. Conclusions

In this study, a Fe_3_O_4_/PVA composite was successfully synthesized by the precipitation process to degrade Methyl Orange using a laboratory photocatalytic reactor.

The FTIR spectra showed that the PVA was successfully added onto the Fe_3_O_4_ particles’ surface; the presence of a peak at ~530 cm^−1^ in the obtained composites (Fe_3_O_4_ and Fe_3_O_4_/PVA) was assigned to the stretching vibrations of the Fe-O bonds. The peaks observed at 1388 cm^−1^ (Fe_3_O_4_/PVA) and 1324 cm^−1^ (PVA) were assigned to methylene stretching. The formation of the magnetite phase was definitively confirmed by XRD. The surface morphology showed that the particles had an uneven distribution with different shapes and sizes. The micrographs indicated that the Fe_3_O_4_ particles had been stabilized by adding PVA to their surface and the Fe_3_O_4_/PVA particles had a relative diameter of about 11 nm.

The photodegradation experiments indicated that the higher Methyl Orange removal efficiency of 98.65% was obtained for Fe_3_O_4_/PVA. This higher removal efficiency of the sample indicates that an almost complete decomposition of Methyl Orange occurred. The photodegradation of dye improved rapidly by increasing the amount of H_2_O_2_. The dye removal efficiency of the Fe_3_O_4_/PVA was over 78% with 120 min of photodegradation at values of pH of 2 and 4 in comparison to the photodegradation at a pH of 3 where the dye removal was 99.3%.

The chemical and structural properties and high photocatalytic activity of Fe_3_O_4_/PVA indicated that the prepared composite can also be used in the adsorption or photodegradation processes of other dyes from different wastewater.

## Figures and Tables

**Table 1:** Dye removal percentage values for composite at different amounts of composite.

Amount of Composite, g	Fe_3_O_4_	Fe_3_O_4_/PVA
	Dye Removal, %	
1.5	65.89 ± 0.05	78.32 ± 0.03
2	77.56 ± 0.07	89.25 ± 0.04
3	87.71 ± 0.04	92.54 ± 0.04
5	91.37 ± 0.04	98.65 ± 0.03

**Table 2:** Dye removal percentage values for composite at different amounts of hydrogen peroxide (H_2_O_2_).

Amount of H_2_O_2_, mL	Fe_3_O_4_	Fe_3_O_4_/PVA
	Dye Removal, %	
3.5	58.41 ± 0.06	60.62 ± 0.04
6.7	71.77 ± 0.03	80.78 ± 0.05
10	67.83 ± 0.04	75.14 ± 0.06

## Data Availability

Not applicable.
